# Snake bite in India: A few matters to note

**DOI:** 10.1016/j.toxrep.2018.08.010

**Published:** 2018-08-17

**Authors:** Siju V. Abraham

**Affiliations:** Department of Emergency Medicine, Jubilee Mission Hospital, Medical College & Research Institute, Jubilee Mission P. O., Thrissur 680 005, Kerala, India

Sir,

This in reference to; the article by Ghosh R et al. A retrospective study of clinico-epidemiological profile of snakebite related deaths at a Tertiary care hospital in Midnapore, West Bengal, India published in the Toxicology reports. 2018 Dec 31;5:1–5. The effort taken to quantify the magnitude of this neglected tropical disease in Midnapore is to be appreciated [1].

The graphical abstract, published along with the article is not essentially something that is applicable in the Indian sub continent.

There are three matters I would like to bring to the attention of readers in this aspect,

1 Brown snakes (Peudonaja textilis) and Tiger snake (Notechis sculatus) are medically important snakes in the Australian subcontinent and their antivenom is not used, nor is it needed in India [2]. The polyvalent antivenom used in India contains antibody to neutralise Common Krait(Bungarus caeruleus), Cobra (Naja naja), Saw scaled viper (Echis carinatus) and Russells viper (Daboia russelli) [3]. There are other medically important snakes in India, like the Hump Nosed Pit viper (Hypnale hypnale) and Malabar Pit viper (Trimeresurus malabaricus) for which there are no antivenom available [3].
2 20′WBCT (20 min Whole Blood Clotting Test) is whole blood clotting 'test' and not time [3]. 20′WBCT is a bed side coagulation study in which a blood sample is kept in a clean glass vial or tube undisturbed for a period of 20 min and tilted to see if it has clotted or not. If it has not clotted it is taken as an 'abnormal' 20′WBCT. You get a binary answer, 'clotted' or 'not clotted' instead of a quantification of the time to form a clot. The reliability of the test even though is questionable, it is an unparalleled cost-effective bed side coagulation study [4].
3 VDK (venom detection kit) mentioned in the Graphical abstract [1], is an unmet need in the Indian subcontinent. Over the years various assays and diagnostic tests based on various principles have been carried out in an attempt to develop an effective, reliable, sensitive, cost-effective, and portable VDK [[5], [6], [7], [8]]. Although there have been several reports on venom detection and assay protocol development from various part of the world including India, most still remains as experimental model and is not available as field detection kits [9]. VDK has been developed and successfully applied to detect envenomation by common venomous snakes of Australia, and helps in administration of the appropriate monovalent antivenom to the affected patients [10].

Though Australia has more deadly snakes than the Indian subcontinent, the mortality due to snake bite in Australia averages less than 5 per annum, whereas in India over 50,000 deaths are estimated per annum [11,12].

Development of a region specific VDK, better screening tool than WBCT, better quality antivenoms and snake bite treatment protocols are paramount along with public and health care personnel education with regards to the same.
